# Supplementary material for: Hot carriers from intra- and interband transitions in gold-silver alloy nanoparticles
Source: Commun Chem. 2024 Aug 1;7:169. doi: 10.1038/s42004-024-01244-w (PMC11294548; doi:10.1038/s42004-024-01244-w)
Supplement: Supplementary file 1 — Supplementary information [file 42004_2024_1244_MOESM1_ESM.pdf]

Supplementary Information

Hot Carriers from Intra- and Interband Transitions in Gold-Silver Alloy Nanoparticles

Shreyas Ramachandran,† Simão M. João,† Hanwen Jin,† and Johannes Lischner*,†,‡

†*Department of Materials, Imperial College London, South Kensington Campus, London SW7 2AZ, United Kingdom*

‡*The Thomas Young Centre for Theory and Simulation of Materials, London E1 4NS, United Kingdom*

E-mail: j.lischner@imperial.ac.uk

Computational details

Following Jin and coworkers,[1] the hot-electron generation rate $N_e(E,\omega)$ is expressed as

$$N_e(E,\omega) = \frac{4\pi}{\hbar V}\int_{-\infty}^{\infty} d\mathcal{E}'\delta(E-\mathcal{E}';\sigma)\int_{-\infty}^{\infty} d\mathcal{E}\phi(\mathcal{E},\mathcal{E}',\omega)\delta(\mathcal{E}-\mathcal{E}'-\hbar\omega;\gamma)f(\mathcal{E})(1-f(\mathcal{E}')), \quad (1)$$

$$\phi(\mathcal{E},\mathcal{E}',\omega) = \sum_{if}\left|\langle f|\hat{\Phi}_{tot}(\omega)|i\rangle\right|^2\delta(\mathcal{E}-E_i)\delta(\mathcal{E}'-E_f).$$

$\phi(\mathcal{E},\mathcal{E}',\omega)$ can be obtained from the trace of the operator $\delta(\mathcal{E}-\hat{H})\hat{\Phi}_{tot}(\omega)\delta(\mathcal{E}'-\hat{H})\hat{\Phi}_{tot}(\omega)$. To calculate the trace using the kernel polynomial method, the energy variables ($\mathcal{E}(\mathcal{E}') \mapsto \varepsilon(\varepsilon')$) and Hamiltonian ($\hat{H} \mapsto \hat{h}$) need to be scaled and shifted such that the spectral weight

of the operator is in the interval [-1,1]. In this interval, Chebyshev polynomials of the first kind $T_n(\varepsilon) = \cos(n \arccos \varepsilon)$ form a complete set of orthogonal functions.

The spectral operator for hot-carrier generation rates can now be expressed as $\delta(\varepsilon - \hat{h}) = [2/(\pi\sqrt{1-\varepsilon^2})] \sum_{n=0}^{\infty}(1+\delta_{n0})^{-1}T_n(\hat{h})T_n(\varepsilon)$. In numerical calculations, this series is truncated after N terms. In the truncated series, each term is multiplied with a coefficient of Jackson's kernel[2] which effectively replaces a delta function with a Gaussian of width $\sigma = \pi/N$. Inserting the coefficients into $\phi(\varepsilon, \varepsilon', \omega)$ yields

$$\phi(\varepsilon, \varepsilon', \omega) \approx \frac{1}{E_-^2} \sum_{n=0}^{N-1} \sum_{m=0}^{N-1} \frac{4\mu_{mn}(\omega) T_m(\varepsilon) T_n(\varepsilon')}{\pi^2 \sqrt{(1-\varepsilon^2)(1-\varepsilon'^2)}} \times \frac{J(n,N)J(m,N)}{(1+\delta_{n0})(1+\delta_{m0})}, \tag{2}$$

where the Chebyshev moments are given by $\mu_{mn}(\omega) = \mathrm{Tr}[T_m(\hat{h})\hat{\Phi}_{tot}(\omega)T_n(\hat{h})\hat{\Phi}_{tot}(\omega)]$. The trace is calculated using a stochastic approach that scales linearly with the system size. Specifically, the moments are expressed as

$$\mu_{mn}(\omega) \approx \left| eE_0 \frac{3\epsilon_m}{2\epsilon_m + \epsilon(\omega)} \right|^2 \sum_{k=1}^{K} \langle k | T_m(\hat{h}) \hat{z} T_n(\hat{h}) | k \rangle,$$

where k is a random vector and K represents the total number of random vectors. In our calculations, we use 2000 Chebyshev polynomials and 10 random vectors. Once the moments are obtained, the hot-carrier generation rates can be calculated with Eq. 1 and 2 and the energies are rescaled back to the original energy interval ($\varepsilon(\varepsilon') \mapsto \mathcal{E}(\mathcal{E}')$).

For each alloy composition, we calculate hot-carrier generation rates for 100 different disorder realizations and then average the results. Fig. 1 shows that the resulting hot-carrier generation rates are well converged.

Figure 2 shows the band structure of bulk crystalline Au. At high energies above the Fermi level (approx. 3.0 eV), we observe the onset of an additional band which gives rise to a small peak in the density of states. This band gives rise to additional interband transitions from the sp-band if the photon energy is larger than 3.0 eV. In Ag, the onset of this band is at approximately 3.4 eV.

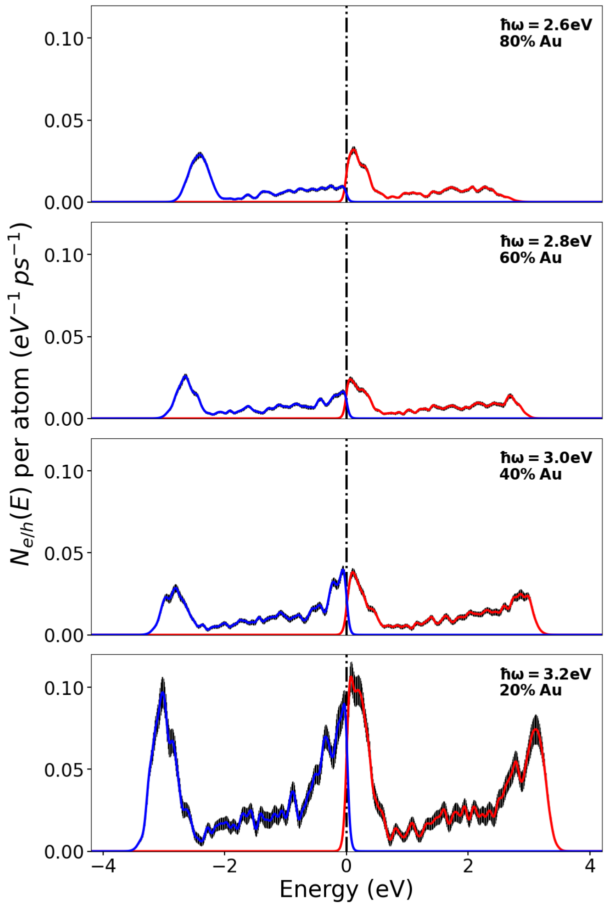

Supplementary Figure 1: Hot-carrier generation rates per atom for spherical Ag-Au alloy nanoparticles of 8 nm diameter. For each alloy composition, the result is shown for the frequency which gives rise to the highest generation rate, i.e. the frequency closest to the localized surface plasmon frequency. The error bars show the variance after averaging over 100 calculations with each calculation considering a different disorder realization. The Fermi level is set to 0 eV.

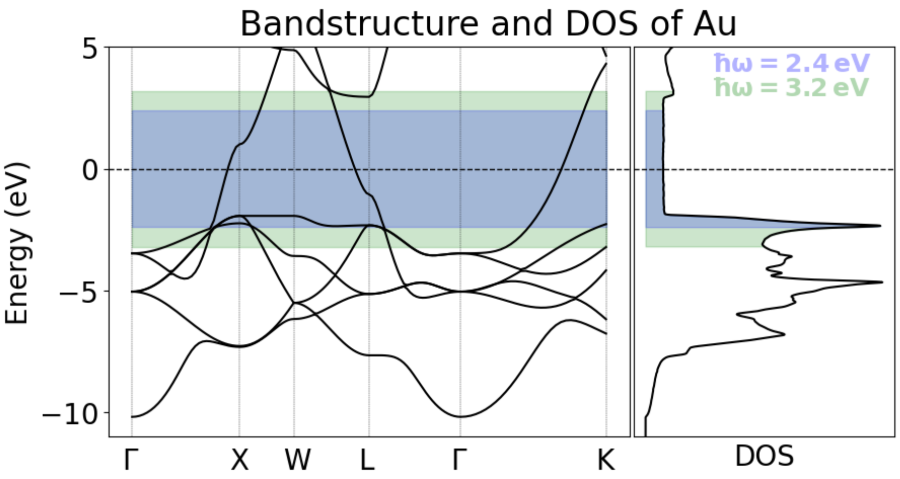

Supplementary Figure 2: Band structure and density of states of Au. The blue (green) shaded area indicates the states that can be accessed by photons of energy 2.4 eV (3.2 eV). The dashed line indicates the Fermi level which is set to 0 eV.

References

(1) Jin, H.; Kahk, J. M.; Papaconstantopoulos, D. A.; Ferreira, A.; Lischner, J. Plasmon-Induced Hot Carriers from Interband and Intraband Transitions in Large Noble Metal Nanoparticles. *PRX Energy* **2022**, *1*, 013006.

(2) Jackson, D. *Über die Genauigkeit der Annäherung stetiger Funktionen durch ganze rationale Funktionen gegebenen Grades und trigonometrische Summen gegebener Ordnung*; Dieterich: Göttingen, 1911.
